# Evaluation of Lipid Extraction Protocols for Untargeted Analysis of Mouse Tissue Lipidome

**DOI:** 10.3390/metabo13091002

**Published:** 2023-09-09

**Authors:** Ashraf M. Omar, Qibin Zhang

**Affiliations:** 1 Center for Translational Biomedical Research, University of North Carolina at Greensboro, North Carolina Research Campus, Kannapolis, NC 28081, USA; amalsagheer@uncg.edu; 2 Department of Chemistry & Biochemistry, University of North Carolina at Greensboro, Greensboro, NC 27402, USA

**Keywords:** untargeted lipidomics, lipid extraction, mouse lipidome, mouse tissue, UHPLC-HRMS

## Abstract

Lipidomics refers to the full characterization of lipids present within a cell, tissue, organism, or biological system. One of the bottlenecks affecting reliable lipidomic analysis is the extraction of lipids from biological samples. An ideal extraction method should have a maximum lipid recovery and the ability to extract a broad range of lipid classes with acceptable reproducibility. The most common lipid extraction relies on either protein precipitation (monophasic methods) or liquid–liquid partitioning (bi- or triphasic methods). In this study, three monophasic extraction systems, isopropanol (IPA), MeOH/MTBE/CHCl_3_ (MMC), and EtOAc/EtOH (EE), alongside three biphasic extraction methods, Folch, butanol/MeOH/heptane/EtOAc (BUME), and MeOH/MTBE (MTBE), were evaluated for their performance in characterization of the mouse lipidome of six different tissue types, including pancreas, spleen, liver, brain, small intestine, and plasma. Sixteen lipid classes were investigated in this study using reversed-phase liquid chromatography/mass spectrometry. Results showed that all extraction methods had comparable recoveries for all tested lipid classes except lysophosphatidylcholines, lysophosphatidylethanolamines, acyl carnitines, sphingomyelines, and sphingosines. The recoveries of these classes were significantly lower with the MTBE method, which could be compensated by the addition of stable isotope-labeled internal standards prior to lipid extraction. Moreover, IPA and EE methods showed poor reproducibility in extracting lipids from most tested tissues. In general, Folch is the optimum method in terms of efficacy and reproducibility for extracting mouse pancreas, spleen, brain, and plasma. However, MMC and BUME methods are more favored when extracting mouse liver or intestine.

## 1. Introduction

Lipids are diverse in structure and play critical roles in several metabolic activities, such as energy storage, cell signaling, membrane trafficking, and biosynthetic pathways [1]. Lipidomics refers to the full characterization of lipids present within a cell, tissue, organism, and/or biological system [2]. Lipidomics has gained attention for biomarkers discovery because fluctuations in the lipidome have been reported in various disease conditions such as cancer, diabetes, and cardiovascular diseases [3,4,5,6]. Ultrahigh-performance liquid chromatography-high resolution mass spectrometry (UHPLC-HRMS) has been widely used for untargeted lipidomic analysis with improved sensitivity and specificity [7]. However, one of the bottlenecks affecting this approach is the extraction of lipid content from biological samples. An ideal extraction method should have a maximum lipid recovery and the ability to extract a broad range of lipid classes having different polarities with acceptable reproducibility.

To date, research labs and core facilities use several lipid extraction methods. Lipid extraction usually involves liquid–liquid partitioning using two or more immiscible aqueous and organic solvents (bi- or tri-phasic extraction), or protein precipitation, by using one or more miscible organic solvents (monophasic extraction) [8]. The Folch or Bligh–Dyer methods are the first developed protocols for the sole purpose of extracting lipids. They only differ in the used CHCl_3_-to-MeOH ratios, being 2:1 in the former and 1:2 in the latter, before being partitioned against water [9,10]. Both methods suffer from the use of the hazardous CHCl_3_ in addition to the limitation in high-throughput analysis due to the difficulty of collecting the lower organic layer, which is often contaminated with the carry-over of water-soluble impurities. Despite these drawbacks, for decades, these two methods stood as the “gold standards” for extracting lipids. The past years witnessed the emergence of a few biphasic methods trying to overcome the issues in Folch and Bligh–Dyer methods. The BUME and MTBE (Matyash) methods were developed using 1-butanol (BuOH) or methyl-*t*-butyl ether (MTBE), respectively, instead of CHCl_3_ [11,12]. This made the organic phase become the top layer after liquid–liquid partitioning against water and, therefore, less contaminated with water-soluble impurities and easily automated for the preparation of a large number of samples. However, BuOH has a high boiling point and, therefore, is more prone to cause lipid hydrolysis during the lengthy evaporation process. Moreover, MTBE is a more polar solvent compared to CHCl_3_ and, therefore, has a higher capacity for solubilizing water (1.4%) and may carry over water-soluble contaminants [8]. Lipid extraction was also performed by using single or multiple miscible organic solvent(s), causing protein precipitation prior to centrifugation and collecting supernatants. Among the solvent(s) used for the monophasic extraction are MeOH [13,14], EtOH [13], isopropyl alcohol (IPA) [13,14,15], ACN [14], n-hexane [16], MeOH/MTBE/CHCl_3_ (MMC) [17], hexane/IPA [18], and EtOAc/EtOH (EE) [19]. The advantages of using monophasic solvents include being fast, cheap, less complex, and more feasible for high throughput and automation. However, the lipid extracts collected using these methods usually contain salts and other polar metabolites and, hence, are less clean compared to the biphasic extracts [20].

Several evaluations have been carried out comparing the effectiveness and reliability of different lipid extraction methods [14,18,20,21,22,23]. Rais et al. [18] compared Folch, Bligh–Dyer, and MTBE biphasic methods together with the acidified Bligh–Dyer and a hexane-IPA monophasic method for their ability to extract lipids from human low-density lipoprotein [18]. Results showed that the recoveries of the major lipid classes, such as triglycerides (TG), cholesteryl esters (CE), and phosphatidyl cholines (PC), were similar. However, for less abundant classes, such as phosphatidyl inositols (PI), lysophosphatidyl cholines (LPC), ceramides (Cer), and cholesteryl sulfates, there was a significant variation between methods. The Folch method was the most effective for extraction of a broad range of lipid classes, although the hexane–isopropanol method was best for non-polar lipids, and the MTBE method was suitable for lactosyl ceramides (LacCer) with no single method showing optimal performance for all lipid classes [18]. Moreover, Zhang et al. [21] evaluated and optimized lipid extraction from PANC-1 human pancreatic cancer cell lines using Bligh–Dyer, MTBE, MMC, and hexane-IPA methods. Results demonstrated that the MTBE method provided a better extraction efficiency for different lipid classes [21]. Furthermore, Wong et al. evaluated Folch, MTBE, and MeOH/BuOH (Alshehry) methods for lipid extraction from human plasma and found that the Alshehry method was as effective as Folch and MTBE in extracting most lipid classes and was more effective in the extraction of polar lipids [24]. In addition, Horing et al. [20] carried out a thorough evaluation of monophasic lipid extraction methods, including MeOH, EtOH, IPA, BuOH, ACN, BuOH/MeOH (3:1), and MeOH/ACN against the Bligh–Dyer method for extracting lipids from human plasma. The study concluded that the recovery of polar lysophospholipids was sufficient for all tested solvents. However, non-polar lipid classes such as TG and CE had dramatically lower extraction efficiencies when extracted with EtOH, MeOH, MeOH/ACN, or ACN [20]. 

Most of the literature comparing lipid extraction methods had contradictory conclusions based on the sample matrix and the use of Shotgun or LC-MS approaches. This is understandable since Shotgun lipidomics requires a high degree of sample preparation to ensure the removal of unwanted components, such as salts and polar metabolites, which may suppress lipid ions during electrospray ionization (ESI). In contrast, lipidomic approaches utilizing reversed phase LC separation prior to MS analysis require less stringent sample preparation as polar impurities are eluted at the solvent front and do not interfere with the MS analysis of the lipids [25]. It is worthy to mention that few of the reported evaluations had been made using mouse-derived samples [11,26]. In addition, most of the reported comparisons had focused on extracting stable isotope labeled (SIL) standards spiked to the investigated sample matrix without studying the effect on the endogenous lipidome as a whole. Moreover, the sample matrix could affect the extraction results due to the difference in the abundances of different lipid classes. Therefore, in this study, three monophasic extraction methods, IPA, MMC, and EtOAc/EtOH (EE), as well as three biphasic protocols, Folch, BUME, and MTBE, were evaluated for extracting the mouse lipidome of six different tissues—pancreas, spleen, liver, brain, plasma, and small intestine, representing different cellular architecture and lipid composition. 

## 2. Materials and Methods

### 2.1. Chemicals and Reagents

IPA, MeOH, 1-BuOH, heptane, formic acid, and H_2_O were purchased from ThermoFisher Scientific (Waltham, MA, USA). EtOAc was obtained from VWR (Randor, PA, USA). MTBE was purchased from Sigma–Aldrich (St. Louis, MO, USA). All solvents were HPLC grade or higher. Ammonium formate was obtained from Optima (Douglas, GA, USA). The composition of the used stable isotope-labeled internal standard (SIL-ISTD) mixture is listed in Table 1. ISTD belonging to (lyso)PC, (lyso)PE, phosphatidyl glycerol (PG), CE, sphingomyelins (SM), monoglycerides (MG), diglycerides (DG), and TG classes were obtained as a mixture (Splash) from Avanti Polar Lipids (Alabaster, AL, USA). AcCa, acylethanolamines (AEA), Sph, Cer, and coenzyme Q (CoQ) standards were purchased from Cayman (Ann Arbor, MI, USA), while the GlcCer standard was obtained from Avanti.

### 2.2. Mouse Tissue Samples

Mouse tissues were provided by the Jackson Laboratory (Bar Harbor, ME, USA) and harvested from a female BALB/c mouse (Strain # 000651) at 8 weeks of age.

### 2.3. Tissue Homogenization

A small piece of approximately 60–70 mg was cut off a frozen tissue and transferred in a homogenization tube with ceramic beads (V = 2 mL). Samples were suspended in cold aqueous ammonium formate (5 mM) at a concentration of 0.05 mg/µL. The sample was directly homogenized in a Precellys^®^ 24 tissue homogenizer supplemented by a Crylolys Evolution from Bertin Instruments (Berlin, Germany). The homogenizer was operated at 4 °C, 8000 rpm, two cycles of 30 s run time, and a 15 s break interval between cycles [27].

### 2.4. Lipid Extraction Methods

Six extraction methods were evaluated: IPA, MMC, EE, Folch, BUME, and MTBE. Each tissue was extracted in triplets using each method.

#### 2.4.1. The IPA Method

Homogenate (40 mL, representing 2 mg wet tissue) or plasma (10 mL) was mixed with IPA (700 µL), containing 2.5 mL ISTD, in 1.5 mL Eppendorf tubes. The tubes were then vortexed for 5–10 s, followed by shaking on an Eppendorf thermomixer for 1 h at 4 °C. Samples were then centrifuged at 17,500× *g*, 4 °C for 5 min, followed by collecting of the supernatant [15].

#### 2.4.2. The MMC Method

Homogenate (40 mL, representing 2 mg wet tissue) or plasma (10 mL) was mixed with 700 µL MeOH/MTBE/CHCl_3_ (4:3:3), containing 2.5 mL ISTD, in 1.5 mL Eppendorf tubes. The tubes were then vortexed for 5–10 s, followed by shaking on an Eppendorf thermomixer for 1 h at 4 °C. Samples were then centrifuged at 17,500× *g*, 4 °C for 5 min, followed by collecting of the supernatant [17].

#### 2.4.3. The EE Method

Homogenate (40 mL, representing 2 mg wet tissue) or plasma (10 mL) was mixed with 700 µL EtOAc/EtOH (1:1), containing 2.5 mL ISTD, in 1.5 mL Eppendorf tubes. The tubes were then vortexed for 5–10 s, followed by shaking on an Eppendorf thermomixer for 1 h at 4 °C. Samples were then centrifuged at 17,500× *g*, 4 °C for 5 min, followed by collecting of the supernatant [19].

#### 2.4.4. The Folch Method

Homogenate (40 mL, representing 2 mg wet tissue) or plasma (10 mL) was mixed with 700 µL CHCl_3_/MeOH (2:1), containing 2.5 mL ISTD, in 1.5 mL Eppendorf tubes. The tubes were then vortexed for 5–10 s, followed by shaking on an Eppendorf thermomixer for 1 h at 4 °C. This is followed by the addition of 200 µL water, inducing the phase separation of two liquid phases. The tubes were vigorously vortexed for 10 s and centrifugated, at 17,500× *g*, 4 °C for 5 min, followed by the collection of the lower chloroform phase [18].

#### 2.4.5. The BUME Method

Homogenate (40 mL, representing 2 mg wet tissue) or plasma (10 mL) was mixed with 350 µL BuOH/MeOH (3:1), containing 2.5 mL ISTD, in 1.5 mL Eppendorf tubes. The tubes were then vortexed for 10 s, followed by the addition of 350 µL heptane/EtOAc (3:1) and vortexing again shaken for 10 s. Afterward, tubes were shaken on an Eppendorf thermomixer for 1 h at 4 °C. This is followed by the addition of 200 µL acetic acid (1%), inducing the phase separation of two liquid phases. The tubes were vigorously vortexed for 10 s and centrifugated at 17,500× *g*, 4 °C for 5 min, followed by the collection of the upper organic phase [11].

#### 2.4.6. The MTBE (Matyash) Method

Homogenate (40 mL, representing 2 mg wet tissue) or plasma (10 mL) was mixed with 700 µL MTBE/MeOH (2:1), containing 2.5 mL ISTD, in 1.5 mL Eppendorf tubes. The tubes were then vortexed for 10 s. This is followed by the addition of 200 µL water, inducing the phase separation of two liquid phases. The tubes were vigorously vortexed for 10 s and centrifugated at 17,500× *g*, 4 °C for 5 min, followed by the collection of the upper organic phase [18].

The supernatants or organic phases collected using any of the aforementioned methods were then dried under a stream of nitrogen using a Biotage Turbovap LV (Charlotte, NC, USA) and stored at −80 °C until analyzed by LC-MS. Those lipid extracts served as samples spiked with the ISTD “pre-extraction”. Parallel samples were extracted using the same elaborated methodology but without spiking the ISTD. The latter samples were spiked with ISTD immediately prior to the LC-MS analysis (check 2.6) and served as samples spiked with the ISTD “post-extraction”.

### 2.5. LC-MS Analysis

Extracted lipid samples were resuspended in 60 µL of ACN/IPA/H_2_O (65:30:5, *v*/*v*/*v*) and transferred into autosampler vials containing a 300 µL glass insert. Lipid extracts were subjected to LC-MS analysis using a Vanquish Horizon UHPLC (ThermoFisher Scientific; Waltham, MA, USA) coupled to a Q Exactive HF mass spectrometer (ThermoFisher Scientific; Waltham, MA, USA), using data-dependent MS/MS acquisition. An Accucore Vanquish C18+ column (150 × 2.1 mm, 1.5 μm) with 2.1 mm ID filter cartridge (0.2 um) was used at a flow rate of 230 µL/min, and the following gradient: 0.0–1.0 min (10% B), 2.0 min (30% B), 3.5 min (50% B), 7.0 min (60% B), 17.0 min (70% B), 18.0 min (80% B), 20.0 min (95% B), 22.0–28.0 min (100% B), and 28.1–32.0 min (10% B). Prior to each injection, the LC column was equilibrated for 5 min at 10% solvent B. Solvents A and B consisted of ACN/H_2_O (1:1, *v*/*v*) and IPA/ACN/H_2_O (88:10:2. *v*/*v*/*v*), respectively, both containing 10 mM ammonium formate and 0.1% formic acid. The quality controls (QC) were generated by pooling 5 μL of each sample belonging to the same tissue type and were injected intermittently during the whole process of sample injections to evaluate the reproducibility and stability of the LC-MS system. The following parameters were set for the mass spectrometer: 20 Arb, 1 Arb, 250 °C, 300 °C, and 50% for sheath gas, sweep gas, ion transfer tube, vaporizer temperature, and S-lens RF level, respectively. The aux gas was set at 5 and 7 Arb in positive and negative ion modes, respectively. The ion source was operated using heated ESI with an ion spray voltage set at 3500 and 3000 V in positive and negative ion modes, respectively. Mass spectra were acquired in the scan range of 250–1400 *m*/*z* at a mass resolution of 120 k (at 200 *m*/*z*), followed by data-dependent MS/MS with a mass resolution of 30 k (at 200 *m*/*z*) for the 10 most abundant ions. The AGC targets were 1 × 10^6^ and 1 × 10^5^ in full-scan MS and dd-MS^2^, respectively. The maximum injection time (IT) was set at 75 ms for both full-scan MS and dd-MS^2^. A dynamic exclusion of 8 s and an isolation window of 1.0 *m*/*z* were used when acquiring the dd-MS^2^ data. Moreover, a step collision energy (NCE) of 30 for positive mode and 20, 30, and 40 for negative mode was used when collecting the dd-MS^2^ data.

### 2.6. Lipidomics Data Processing

Lipid identification was performed by LipidSearch 5.0 software (Thermo Scientific, San Jose, CA, USA). During the LC-MS product search, the precursor and product tolerances were set at 10 and 15 ppm mass windows, respectively. During the LC-MS alignment, the RT tolerance and RT correction tolerance were set at 0.05 and 0.5 min, respectively. The S/N, intensity ratio, and valid peak rate thresholds were set at 3.0, 1.5, and 0.5, respectively. The identified lipid hits were filtered according to the “Grades”. TG/DG/AcCa lipids were accepted when identified only with Grades A or B. [M+H]^+^, [M+NH4]^+^ adduct ions were considered precursor ions in positive ion mode, and [M-H]^−^ was considered as a precursor ion in negative ion mode. The identified lipids information (molecular formula, RT, and ion adduct type) was imported as a transition list to be used by Skyline 22.2.0.351 for the accurate area under the curve (AUC) quantification of each lipid hit. Recovery of ISTD was calculated using Equation (1). AUC values were corrected for ^13^C abundance (Type I correction following the guidelines of Lipidomics Standards Initiative) [28] using Equation (2), as previously described [29,30]. The absolute concentrations of identified endogenous lipids were calculated using Equation (3).

Recovery % = (AUC_*ISTD*,_*_pre-extraction_*/AUC*_ISTD_*_,_*_post-extraction_*) × 100 (1)

where AUC_*ISTD*,_*_pre-extraction_* = area under curve for ISTD spiked before extraction, AUC*_ISTD_*_,_*_post-extraction_* = area under the curve for ISTD spiked after extraction

AUC*_n_*_(*k*)*total*_ = AUC*_n_*_(*k*)_ (1 + 0.0109*n* + 0.0109^2^*n*(*n* − 1)/2) (2)

where AUC*_n(k)total_*= total ion area under curve, AUC*_n(k)_* = quantified area under curve of monoisotopic mass, *n* = No. of Carbon atoms, *k* = No. of double bonds.

C*_lipid_* = (AUC*_lipid_*/AUC*_ISTD_*) × 100 (3)

where C*_lipid_* = Concentration of lipid species, C*_ISTD_* = concentration of ISTD, AUC*_lipid_* = corrected area under curve for lipid species, AUC*_ISTD_* = area under curve for ISTD.

### 2.7. Statistical Analysis

All values were expressed as mean ± SD. Absolute concentrations of identified lipids were further processed using Persus 1.6.14.0 by log transformation followed by the visualization of the difference of mouse lipidomes acquired by different extraction methods using the Principal Component Analysis (PCA). 

## 3. Results

### 3.1. Recovery of ISTD in Extraction

**Figure 1:**
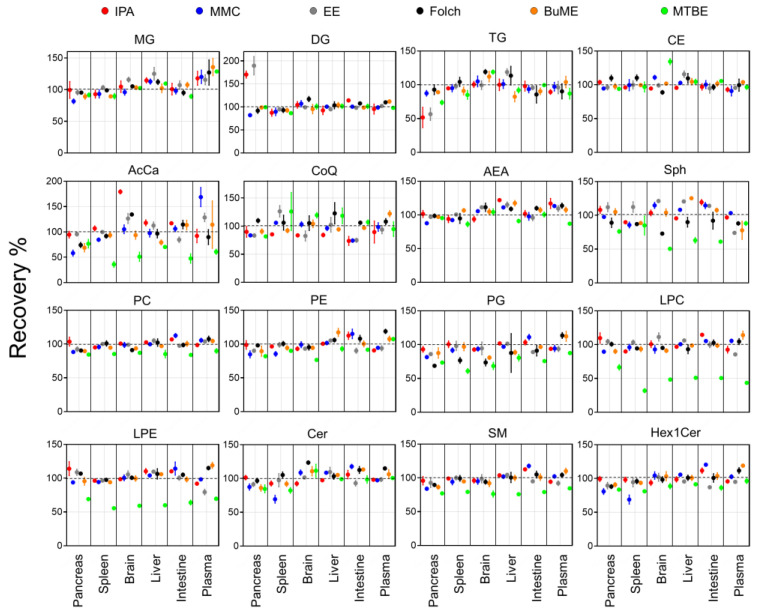
Extraction recoveries of ISTD in mouse tissues by the six tested extraction methods. Displayed are mean and SD (*n* = 3).

**Figure 2:**
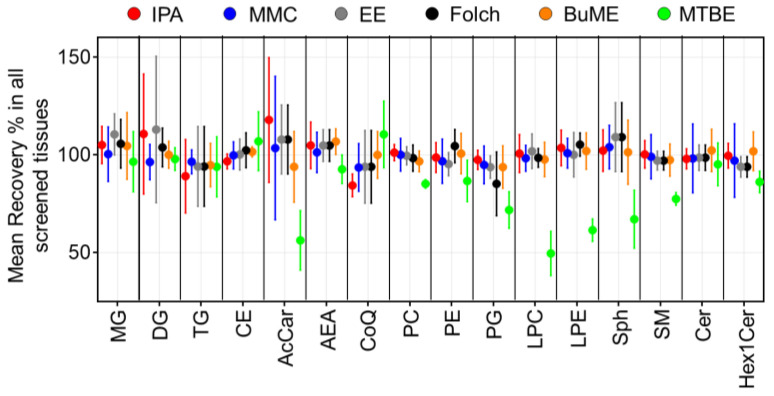
Mean extraction recoveries of ISTD per lipid class. Displayed are mean and SD (*n* = 3).

Recoveries for each method were assessed by comparing the ratio of peak areas of SIL internal standards spiked before and after the extraction of lipids from the six tested mouse tissues (Figure 1). Results showed that the IPA method had an average recovery of 84.4–117.9%. The lowest recovery using IPA was observed when extracting TG from the pancreas (51.7%). Moreover, IPA exerted relatively low recoveries of CoQ from the brain (83.6%), intestine (73.3%), and liver (84.2%). In the case of the MMC method, average recoveries were 93.6–104.0%. The lowest recovery using MMC was observed when extracting AcCa from the pancreas (58.2%). In general, MMC had better recoveries when extracting brain, liver, and plasma, with recoveries ≥92.9%. However, MMC had lower recoveries when extracting pancreas (≥58.2%), spleen (≥68.8%), and intestine (≥74.3%). For the EE method, on average, the recoveries were 93.8–113.0%. Like IPA, the recovery of TG from the pancreas was the lowest (56.5%). In addition, like MMC, the recovery of CoQ from the pancreas and intestine was low (83.3 and 74.9%, respectively). Plasma had the lowest recovery of LPE (79.4%) and Sph (74.0%). As for the Folch method, average recoveries were 85.2–109.7%. The lowest recoveries were observed for PG extracted from the pancreas (68.7%), spleen (76.7%), and brain (73.5%). BUME had average recoveries of 93.8–106.8%. Low recoveries were observed when extracting AcCar from the pancreas (68.8%) and liver (79.3%) in addition to Sph from plasma (77.8%) and TG from the liver (82.4%). Finally, the MTBE method had the lowest average recoveries among all tested methods (49.6–110.5%). Interestingly, MTBE had very low recoveries when extracting PG (71.8% ± 9.3), LPC (49.6% ± 11.3), LPE (61.5 ± 5.7), and AcCar (56.3% ± 15.2). The average recoveries of each lipid class using the tested methods were compared across all mouse tissues (Figure 2). Results showed that, in general, all tested methods had comparable results when extracting non-polar lipids (MG, DG, TG, and CE). However, the recovery of polar lipids (LPC, LPE, Sph, SM, and AcCar) was dramatically reduced when using the MTBE method. Moreover, other polar lipids (PC, PE, PG, and Hex1Cer) had slightly lower recoveries using MTBE.

The coefficient of variations (CV%) of the recoveries calculated for three preparation replicates of each tissue can provide information on the reproducibility of each method in extracting the spiked ISTD. Results showed that, on the one hand, the recovery of polar lipids (PC, PE, LPC, LPE, AEA, SM, Cer, and Hex1Cer) was very reproducible with CV ≤ 10%. On the other hand, the extraction of non-polar lipids did not have the same degree of reproducibility. TG had a CV% of 29.1 when extracted with IPA from the pancreas. Moreover, CoQ had a low reproducibility when extracted with MTBE from the spleen and with IPA from plasma, with CV of 26.9 and 21.8%, respectively.

### 3.2. Characterization of Tissue-Specific Lipidome

#### 3.2.1. Lipid Molecules Identified in Each Tissue

To further compare the efficiency of the six evaluated extraction methods, the endogenous lipidome of the six mouse tissues was identified using the pooled samples of the same tissue type in positive and negative ion modes. Identified lipids were then quantified using a single-point calibration against the spiked ISTD belonging to each examined lipid class. A cut-off threshold was made to involve lipid species in the study only if these lipids were quantified in the QC sample (a pooled sample was periodically injected into the LC-MS system every 20 injections) with a concentration CV ≤ 25%. Details of identified lipids per mouse tissue are listed in Table 2 and Table S1–S6. In total, 546–794 lipids were identified. The highest number of identified lipids was in the spleen (794 lipids), and the lowest number was in brain samples (546 lipids). PC was the predominantly identified class (~30%), followed by PE and TG (each ~14.6%). LPC, LPE, SM, MG, and DG had identification rates of 4.1–7.7%. Moreover, Sph, AEA, and AcCa were minor species with a low identification rate (≤1%).

#### 3.2.2. Concentration of Lipids in Each Tissue

**Figure 3:**
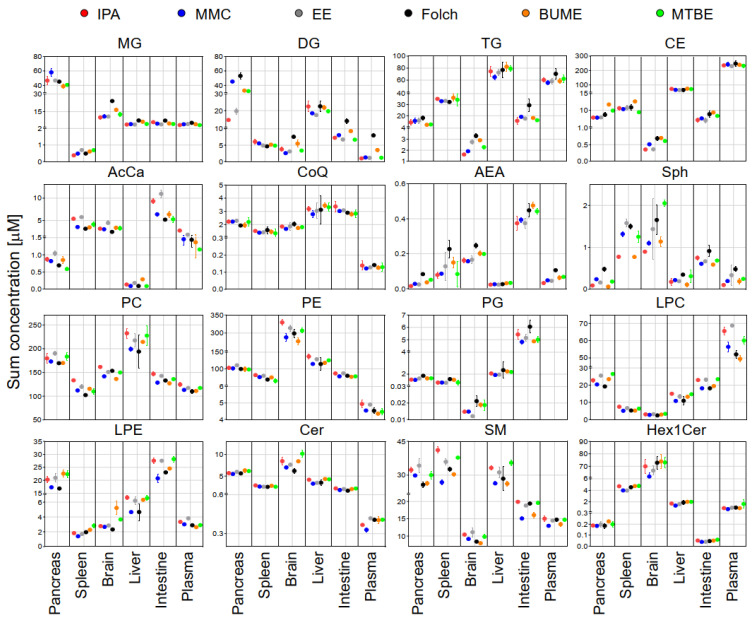
The sum concentrations of identified lipids in each mouse tissue using the six extraction methods. Displayed are mean and SD (*n* = 3).

The sum concentration of identified lipids from the same class in each tissue using different extraction methods is shown in Figure 3. Results exhibited that, except DG and TG, the sum concentrations of identified lipids were comparable across different extraction methods. DG showed slightly higher concentrations in the case of Folch extraction of the pancreas, brain, plasma, and intestine. TG showed slightly higher concentrations in the case of Folch extraction of the pancreas, plasma, and small intestine.

#### 3.2.3. Effect of the Extraction Method on the Fatty Acyl (FA) Chain Length of Mouse Pancreatic Lipidome 

**Figure 4:**
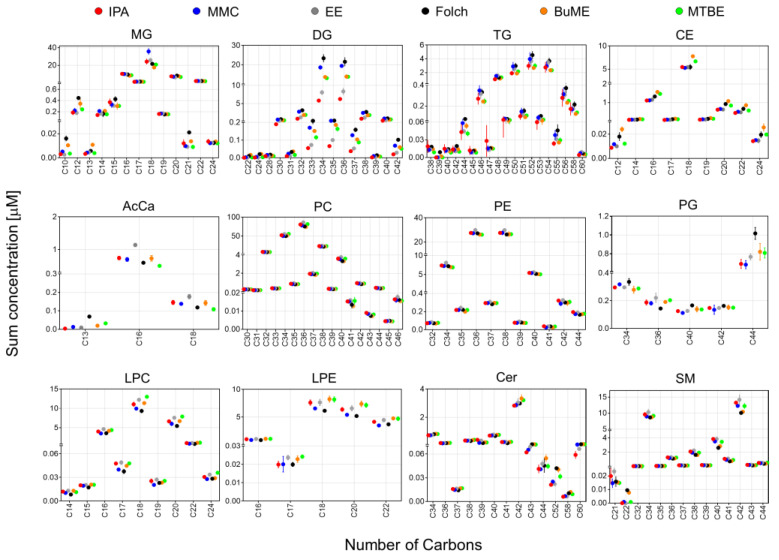
The sum of concentrations of identified lipids in mouse pancreas by the six extraction methods in relation to the total no. of carbons in the fatty acyl side chain(s). Since SM species were identified as the sum composition without providing information on the composition of the sphingoid base, the sum concentrations were plotted against the total no. of carbons rather than the no. of carbons in the fatty acyl chain. Displayed are mean and SD (*n* = 3).

In Figure 4, the sum concentrations of identified lipid species from the mouse pancreas were plotted against the total number of carbons in the FA side chain(s). Since SM species were identified as the sum composition without providing information on the composition of the sphingoid base, only for SM, the sum concentrations were plotted against the total no. of carbons rather than the no. of carbons in the FA chain. Results showed that, except for DG and TG, all extraction methods gave very comparable results using different extraction methods. For DG and TG, the highest abundance was observed when using Folch. For DG, the lowest abundance was observed when using the IPA or EE methods. For the DG species with FA chain having total carbons of 34–37, there was ~two-fold difference in abundance of the DG between that extracted with Folch and with IPA or EE (Figure 4).

### 3.3. Reproducibility of Extraction Methods

#### 3.3.1. Principal Component Analysis (PCA)

**Figure 5:**
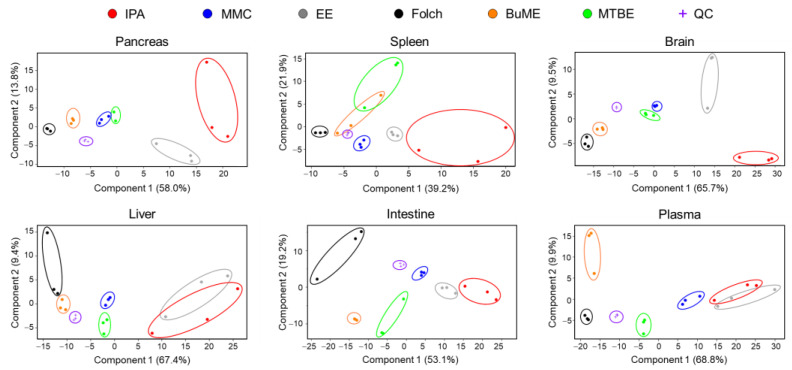
PCA analysis of absolute concentrations of endogenous mouse lipids extracted by different methods.

The overall differences in the lipidome obtained using different extraction protocols were examined using unsupervised principal components analysis (PCA). As shown in Figure 5, the quality control (QC) samples, periodically injected throughout the analysis, were almost superimposed in the PCA plot, demonstrating that all detected lipid species in the QC samples were consistent with no shifts during the entire analysis period, the LC-MS system was stable, and the analysis was repeatable. PCA also showed a clear clustering of samples with the same extraction method and segregation of samples between different extraction methods, indicating that each lipid extraction method generated different lipidomic profiles (Figure 5). In all evaluated tissues, the IPA method had the least clustering, suggesting poor reproducibility. Moreover, EE showed poor reproducibility in extracting lipidomes of the pancreas, brain, liver, and plasma. Folch showed high reproducibility in extracting lipidomes of the pancreas, spleen, brain, and plasma. However, Folch gave poor reproducibility when extracting lipidomes of liver and intestine tissues. MMC, on the other hand, gave high reproducibility except for plasma, for which Folch gave the highest clustering and reproducibility. For the liver and intestine lipidomes, MMC and BUME methods showed the highest reproducibility (Figure 5).

#### 3.3.2. Method Coefficient of Variations (CV)

**Figure 6:**
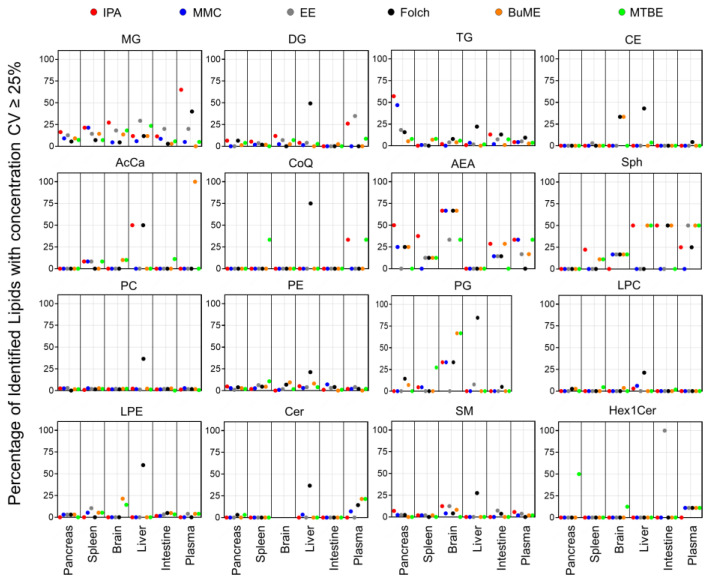
Percentage of identified lipids with absolute concentration CV ≥ 25%.

To check the repeatability of extracting endogenous lipids in greater detail, CV% of the sum concentrations of each lipid class from the three technical replicates prepared for each tissue are shown in Figures S1–S4. Figure 6 presents the percentage of identified lipids with concentrations having CV ≥ 25%. Results showed that the Folch method suffered from poor reproducibility when extracting liver samples with median CV% values dramatically higher than the other extraction methods in almost all identified lipid classes. Apart from that, in general, polar lipid classes (PC, PE, PG, LPC, LPE, SM, Cer, and Hex1Cer) had high reproducibility with a median CV < 15% (Figures S2 and S3). Moreover, the number of polar lipid species with CV ≥ 25% was very low (in most cases <15% of the total number of identified lipids within the same class, Figure 6). The exception to that was the endogenous PG, which, when extracted from the brain using MMC, Folch, BUME, or MTBE methods, had a median CV of 16.6, 24.4, 27.3, or 29.1%, respectively (Figure S2) and >30% of identified lipids had CV ≥ 25% (Figure 6). In addition, when extracting PG lipid species from the spleen using MTBE, sum concentrations had a median CV of 18.4% (Figure S2), and 27.3% of identified PG had CV ≥ 25% (Figure 6). Another exception was Hex1Cer, which had a median CV of 16.9% when extracted using MTBE from the pancreas and 34.2 and 22.6% when extracted from the intestine using EE or MTBE methods, respectively (Figure S3). In addition, EE had bad reproducibility when extracting Hex1Cer from the intestine, as all identified Hex1Cer had CV ≥ 25% (Figure 6). Moreover, CE had a high reproducibility with a median CV < 15% in all tissues and all extraction methods, except when employing Folch for extracting liver samples (Figure S4). A relatively high fraction (33.3%) of CE extracted from the brain using either Folch or BUME had CV ≥ 25% (Figure 6). On the other hand, the reproducibility in extracting glycerolipids was lower than polar lipids. The IPA method had poor reproducibility when extracting MG from plasma and TG from the pancreas with a median CV of 38.9 and 29.4%, respectively (Figure S1), and more than half of those identified lipids had CV ≥ 25% (Figure 6). BUME had the highest reproducibility in extracting MG, DG, and TG from all six tissues with median CV values < 15% (Figure S1). For Sph, AcCa, and CoQ, MMC had the best reproducibility with median CV values < 15% in all tissues (Figure S4), and less than 17% of identified lipids had CV < 25% (Figure 6). For AEA, BUME had the best reproducibility with median CV values ≤ 15% in all mouse tissues (Figure S4). It is worth mentioning that 21.4% of identified Cer from plasma and 50% of identified Hex1Cer from the pancreas, using the MTBE method, had CV ≥ 25%. Moreover, 21.4% of identified LPE from the brain using the BUME method had CV ≥ 25% (Figure 6).

## 4. Discussion

In general, lipidomics applications require sample-preparation methods that are fast, reproducible, and able to extract a wide range of lipids with different polarities. In addition, samples may be available in only limited amounts, posing practical requirements to develop efficient, sample-saving experimental procedures [17]. The aim of this study was to investigate the effectiveness of lipid extraction protocols, namely the IPA [15], MMC [17], EE [19], Folch [18], BUME [11], and MTBE [18] methods. The reasons behind choosing these methods are: First, to cover two categories of lipid extraction, i.e., methods 1 to 3 are monophasic and depend mainly on protein precipitation, methods 4 to 6 are biphasic and rely on the liquid–liquid partitioning between two immiscible phases. Second, to cover a wider range of solvents used in lipid extraction. For example, both Folch and Bligh–Dyer methods use MeOH/CHCl_3_/H_2_O but in different ratios. Therefore, the information we learned from Folch could be applicable to Bligh–Dyer. IPA, EE, and BUME use a set of solvents different from one another. Only MMC (MeOH/MTBE/CHCl_3_) and MTBE (MeOH/MTBE/H_2_O) have solvents in common (MeOH and MTBE). However, CHCl_3_ in the former and H_2_O in the latter differentiate them in terms of being a mono- or bi-phasic extraction method.

A literature survey was conducted to choose the types of sample matrices to be used in this lipid extraction evaluation. Most reported lipid extraction evaluation studies mainly used human plasma, with two studies as an exception, which used human pancreatic cancer cell lines and cerebrospinal fluid samples as matrices [14,18,20,21,22,23,31,32]. Surprisingly, very few reported studies used solid tissues as sample matrices [11,26]. Moreover, in very rare incidents, samples of a non-human origin were used [11,26]. Maybe it has been assumed that the sample type itself is not important in deciding the optimum lipid extraction method, and one method can be “the magic method” for all sample types of plant, animal, or human origins. Therefore, the present study was carried out to test this notion. As a result, this study involved extracting six mouse-derived tissue types which vary in cellular architecture and lipid composition: pancreas, connective and epithelial; spleen, connective and lymphoid; liver, connective and parenchymal; small intestine, connective and muscular; brain, nervous; and plasma, biofluids. Mouse was chosen to be the source of the tested tissues due to a growing number of lipidomics research involving the use of mouse tissues [33,34,35,36] and the availability of tissues in descent amounts, enabling testing the six lipid extraction protocols in replicates. Therefore, the results of the present study could be of use to the lipid community researchers carrying out mouse-related studies.

Prior to the lipid extraction of solid tissues, a homogenization step is needed to physically disrupt tissue structure for effective lipid extraction. Several techniques have been used for tissue homogenization, such as grinding frozen tissue with mortar and pestle [37] or bead-beating-based methods [38,39]. Since homogenization has been evaluated and optimized elsewhere [27,40], in the present study, tissue homogenization was decoupled from the lipid extraction evaluation. The preparation of a fluidic aqueous homogenate of each tissue, followed by fast aliquoting of the homogenate and subsequent lipid extraction of each aliquot, was adopted in the present study. This approach has the following advantages: First, avoid misinterpretation in results due to the zonal distribution of lipid species in certain organs such as the liver [41]. Second, allows straightforward sample handling, enabling testing many extracting methods in parallel using a fixed amount of sample [27]. Third, avoid lipid degradation due to the long exposure of tissues to room temperature during sample weighing [27].

To judge the efficacy of an extraction method, SIL standards are often used. These standards have almost identical structural features to endogenous lipid species but with a slight change in the molecular mass. Therefore, they can be spiked at fixed, known concentrations to the sample matrices. The overall extraction efficacy of a lipid class can be inferred by the recovery of its representative standard(s) [24]. For calculating the recovery, two sets of samples were prepared with SIL standards spiked at two different steps in sample preparation. The first set of samples was spiked with SIL standards after homogenization and prior to lipid extraction, allowing standards possible loss during extraction and further sample preparation procedure. The second set was spiked immediately prior to LC-MS analysis to mimic the situation in which a 100% recovery of standards would have occurred. As suggested by data presented in Figure 1 and Figure 2, SIL standards representing most of the tested lipid classes had comparable recoveries using all tested methods. However, the recoveries of SIL LPC, LPE, SM, Sph, and AcCa standards were significantly lower in the case of the MTBE method. This is consistent with the reported literature [22,24,31,42]. It is interesting that MMC and MTBE, both of which are MTBE/MeOH-based methods, had different recoveries of the aforementioned lipid classes. We reasoned that the low recoveries in the MTBE method are due to the addition of H_2_O; as a result, polar classes have a noticeable solubility in the amphiphilic phase (MeOH/H_2_O), causing the lower recovery of these classes from the lipophilic phase (MTBE/MeOH) [43]. Therefore, the MTBE method should be excluded from being the optimum lipid extraction method.

Using the recovery of spiked SIL standards as the mere parameter for evaluating a lipid extraction method is not comprehensive. That is because that approach depends on using one or few standards to evaluate the overall extraction behavior of a certain lipid class, which is, in fact, comprised of tens or hundreds of species with different structural variables (degree and position of unsaturation, number of carbons, presence of functional groups) and hence a wide range of polarity. Therefore, in the present study, the tissue lipidome itself was identified and quantified to be taken into consideration when evaluating the tested extraction methods in terms of lipid coverage and reproducibility.

For that purpose, pooled samples of lipid extracts of the same tissue type collected using the six tested extraction methods were subjected to LC-MS/MS analysis in positive and negative modes. The identification of endogenous lipid species was carried out using LipidSearch software. For the accurate quantification of endogenous lipids, a single-point calibration was created against the spiked SIL standards prior to extraction as per the guidelines of the Lipidomics Standards Initiative [28]. The sum of absolute concentrations of lipid species of the same class is exhibited in Figure 3. Results showed that except for DG and TG, the sum concentrations of most of the identified lipids were comparable across different extraction methods. This suggested that the addition of SIL internal standards prior to lipid extraction could compensate for the insufficient recovery of polar lipid classes (LPC, LPE, Sph, SM, and AcCar) but not for non-polar lipid classes such as DG and TG. This is consistent with the reported literature [20]. For DG and TG, the highest concentrations in most cases were found using the Folch method. Liver samples had the highest triacylglycerides (TG) abundances when compared with other organs. This is consistent with results reported in the literature [44]. Large variations of DG in the pancreas among different extraction methods may reflect the pancreatic lipase activities or lack of in those solvents.

Lipid species of the same class could have significantly different polarities due to the difference in the number of carbons in the FA side chain(s). To see if a different extract method has a bias against molecular polarities, the pancreatic lipidome was further analyzed to compare the distribution of the abundances of lipid species having different side chain(s) lengths with respect to the tested lipid extraction protocols. It is important to point out that identifying the boundary between abdominal fat and the pancreas in mice can be challenging. The organs were provided by the Jackson Laboratory as frozen tissues and tissue harvesting were handled by experienced personnel there. Moreover, the pancreas was collected from adult mice (8 weeks old, ~20 g), which would facilitate the ability to dissect the boundary between the two tissues. However, that does not rule out the possibility of a minor contamination of the pancreas with some abdominal fat. Results shown in Figure 4 suggest that, except for DG and TG, all extraction methods gave very comparable results. It is interesting to note that for most glycerolipids, glycerophospholipids, and CE, the odd and even number of summed FA chains follow two separate bell-shaped curves. For example, in the case of PC, the total number of carbons in FA side chains starts with either C30 and C31 as the lowest abundant species, reaches the highest abundance at C36 and C37, and after that, the abundance of PC seems to symmetrically decrease until reaching the lowest abundance with PC having C45 and C46 as the total odd and even number of carbons in the FA chain, respectively. The same patterns of symmetry of odd and even FA chains were observed with LPC, LPE, MG, DG, TG, and CE. However, this pattern was not observed with PG, Cer, and SM (Figure 4). For DG and TG, the highest abundance was observed when using Folch, followed by MMC. IPA had the lowest abundance for these two classes. For TG, IPA had the lowest reproducibility (expressed as the high SD error bars) when extracting TG with side chains of C44-C49 as the total number of carbons (Figure 4).

One of the most important aspects when evaluating an extraction method with analytical applications is reproducibility. Developing an extraction protocol with nearly quantitative yields is pointless if it is irreproducible. Therefore, the six tested lipid extraction protocols were evaluated for reproducibility in extracting mouse-derived lipidomes. Judging the reproducibility is usually achieved by comparing the CV% values. In the present study, due to the presence of hundreds of lipids with replicates per method and per screened tissue, it is not an easy task to simply compare CV% values. Therefore, box and whisker plots were generated for the calculated CV% values to aid in the visualization of the distribution and variations of CV% (Figures S1–S4). Additional information was deduced by plotting the fraction (percentage) of lipids having poor CV (≥25%) with respect to the total number of identified lipids (Figure 6). Results suggest that the Folch method has poor reproducibility only when extracting liver samples with CV% dramatically higher than other extraction methods in almost all identified lipid classes. Concomitantly, to have an overall judgment on the repeatability of tested methods, PCA was carried out. The information gathered from investigating the clustering of replicates of the same method gives an idea of the best reproducible method for the overall tissue lipidome. Results depicted in Figure 5 demonstrate that lipidomes generated using the IPA and EE methods had poor reproducibility. Folch, on the other hand, showed high reproducibility in extracting lipidomes of the pancreas, spleen, brain, and plasma. However, it had poor reproducibility when extracting lipidomes from liver and intestine tissues. MMC gave high reproducibility in extracting lipids from all tested tissues except for plasma, for which Folch gave the tightest clustering. For the liver and intestine lipidomes, MMC and BUME methods showed the highest reproducibility.

Among all tested methods, Folch is the oldest and the most well-established. One reason for its popularity is that the combination of MeOH and CHCl_3_ can non-selectively and, in most cases, reproducibly extract most lipid classes from a wide variety of sample matrices. In the Folch method, the addition of MeOH allows the disruption of hydrogen bonding and/or electrostatic networks between the lipid–lipid and/or lipid–protein biomolecules [18,45]. MeOH can also increase the critical micelle concentration and reduce hydrophobic interactions, causing destabilization of micelles by perturbing surface tension [31]. Moreover, CHCl_3_ can extract hydrophobically bound lipids, while water is used for phase separation as well as to decrease the solubility of lipid species in the aqueous phase [45]. While the Folch method has been extensively used for decades and has proven to be effective in many cases, it does have a few limitations, including potential issues with reproducibility in a few reports [22,46]. The reproducibility of any analytical method, including Folch, can be influenced by the specific characteristics of the tissue being analyzed. Liver tissue, in particular, can present some challenges due to its high lipid content and the potential for lipid degradation during the extraction process. To address some of these challenges and improve the reproducibility of lipid extractions, researchers have explored modifications to the CHCl_3_/MeOH/H_2_O lipid extraction methods, such as adding antioxidants or surfactants to prevent lipid oxidation during the extraction process [47,48]. The same principle can be applied to the small intestine tissue, which also could have some limitations during lipid extraction because it is mainly a muscular tissue, and the ability of the extraction system to allow a uniform and superior dispersion of the tissue during extraction would affect the reproducibility of the analytical method. Since, to the best of our knowledge, no reported study in the literature compared the efficiency and reproducibility of lipid extraction from the intestine using different protocols, the present study could provide the first insights on this topic.

## 5. Conclusions

In terms of extraction effectiveness, all tested lipid extraction protocols showed comparable results in extracting most lipid classes. However, the MTBE method showed lower recoveries of LPC, LPE, AcCar, SM, and Sph. Even if the addition of SIL standards could compensate for the lower recovery, the MTBE method should be avoided to recover these species with minor abundance. The IPA and EE methods should also be avoided due to their poor reproducibility. In general, Folch is the optimum method in terms of efficacy and reproducibility of extracting mouse pancreas, spleen, brain, and plasma. However, MMC and BUME methods are more favored when extracting mouse liver or intestine. Of note, the present study demonstrated that lipid extraction reproducibility can be tissue-specific, and if practical, an evaluation should be performed for each tissue type before deciding its optimum lipid extraction protocol.

## Figures and Tables

**Table 1:** Stable isotope-labeled lipid standards used in this study.

ISTD	Conc. (mg/mL) ^1^	ISTD	Conc. (mg/mL) ^1^
PC (15:0/18:1-d7)	6.64	Sph(d16:0)-d7	1.36
PE (15:0/18:1-d7)	0.22	MG (18:1-d7)	0.17
PG (15:0/18:1-d7)	1.26	DG (15:0/18:1-d7)	0.53
LPC (18:1-d7)	1.48	TG (15:0/18:1-d7/15:0)	2.12
LPE (18:1-d7)	0.32	CE (18:1-d7)	16.63
SM (d18:1/18:1-d9)	1.27	AcCa (18:1-d3)	0.97
Cer (d18:1/18:0-d3)	0.73	AEA 16:0-d4	1.37
GlcCer (d18:1-d7/15:0)	0.60	CoQ10-d6	0.48

^1^ the final concentration spiked into the mouse tissue samples injected into the LC-MS.

**Table 2:** No. of identified lipids per lipid class in the six mouse tissues.

Lipid Class	Pancreas	Spleen	Brain	Liver	Intestine	Plasma
MG	55	14	22	17	35	20
DG	77	56	42	75	41	23
TG	77	114	51	123	108	118
CE	24	33	6	28	14	23
PC	199	263	195	181	211	179
PE	106	112	117	99	98	49
PG	14	22	3	13	20	-
LPC	39	44	28	33	55	50
LPE	32	19	14	20	61	25
Sph	1	9	6	2	2	4
SM	43	49	24	40	27	53
Cer	32	28	1	30	9	14
Hex1Cer	2	9	16	6	1	9
AcCa	2	12	10	2	9	10
CoQ	3	3	3	4	3	3
AEA	3	7	8	2	7	4
Total	709	794	546	675	701	584

## Data Availability

Data are contained within the article or Supplementary Materials.

## Supplementary Materials

The following supporting information can be downloaded at https://www.mdpi.com/article/10.3390/metabo13091002/s1, Figure S1: The inter-assay coefficient of variation (CV%) of absolute concentrations of endogenous glycerolipids extracted by different methods; Figure S2: The inter-assay coefficient of variation (CV%) of absolute concentrations of endogenous glycerophospholipids extracted by different methods; Figure S3: The inter-assay coefficient of variation (CV%) of absolute concentrations of endogenous sphingolipids extracted by different methods; Figure S4: The inter-assay coefficient of variation (CV%) of absolute concentrations of endogenous CE, AcCa, AEA, and CoQ extracted by different methods; Table S1: Characterization and quantification of mouse pancreas lipidome using the six tested extraction methods; Table S2: Characterization and quantification of mouse spleen lipidome using the six tested extraction methods; Table S3: Characterization and quantification of mouse brain lipidome using the six tested extraction methods; Table S4: Characterization and quantification of mouse liver lipidome using the six tested extraction methods; Table S5: Characterization and quantification of mouse small intestine lipidome using the six tested extraction methods; Table S6: Characterization and quantification of mouse plasma lipidome using the six tested extraction methods.
