# Supplementary material for: Evaluation of Lipid Extraction Protocols for Untargeted Analysis of Mouse Tissue Lipidome
Source: Metabolites. 2023 Sep 9;13(9):1002. doi: 10.3390/metabo13091002 (PMC10535403; doi:10.3390/metabo13091002)
Supplement: Supplementary file 1 [file metabolites-13-01002-s001.zip › metabolites-2579708-Figures S1-S4.pdf]

Supporting Information

Evaluation of Lipid Extraction Protocols for Untargeted Analysis of Mouse Tissue Lipidome

Ashraf M. Omar[1] and Qibin Zhang[1,2,*]

[1] Center for Translational Biomedical Research, University of North Carolina at Greensboro, North Carolina Research Campus, Kannapolis, NC 28081, USA
[2] Department of Chemistry & Biochemistry, University of North Carolina at Greensboro, Greensboro, NC 27402, USA
[*] Correspondence: q_zhang2@uncg.edu

Table of contents

Figure S1. The inter-assay coefficient of variation (CV%) of absolute concentrations of endogenous glycerolipids extracted by different methods P2

Figure S2. The inter-assay coefficient of variation (CV%) of absolute concentrations of endogenous glycerophospholipids extracted by different methods P3

Figure S3. The inter-assay coefficient of variation (CV%) of absolute concentrations of endogenous sphingolipids extracted by different methods P4

Figure S4. The inter-assay coefficient of variation (CV%) of absolute concentrations of endogenous CE, AcCa, AEA, and CoQ extracted by different methods P5

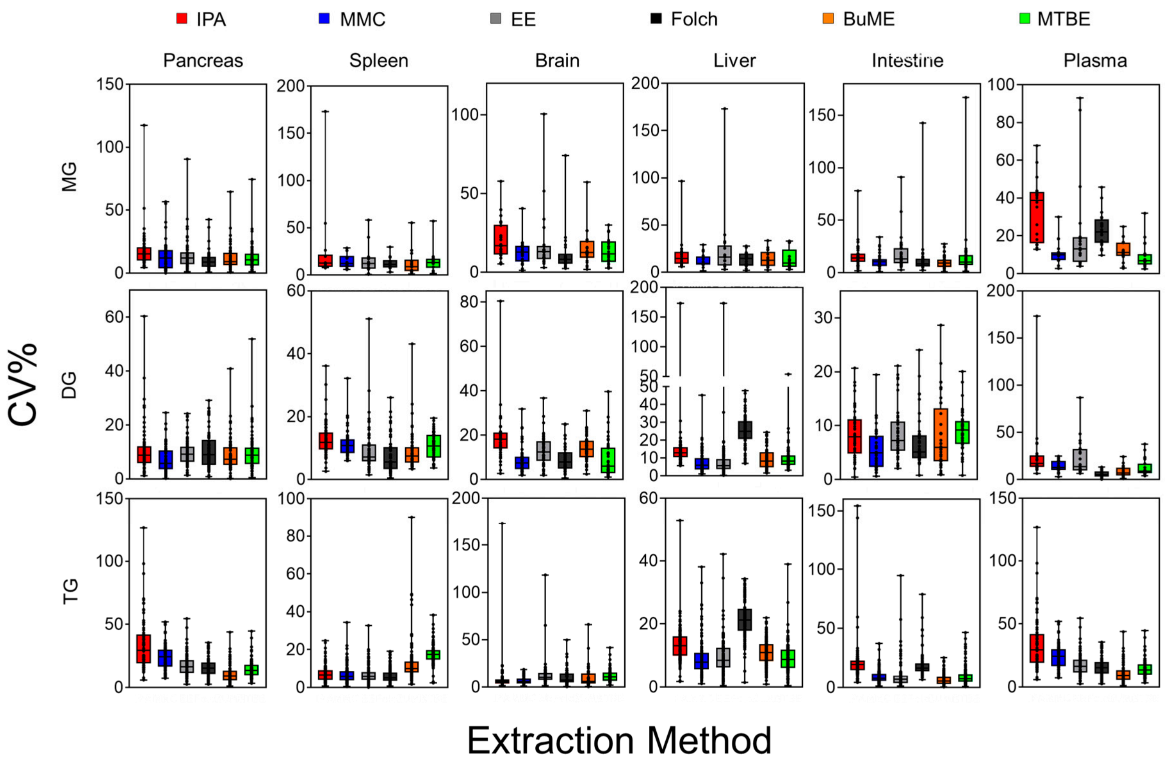

Figure S1. The inter-assay coefficient of variation (CV%) of absolute concentrations of endogenous glycerolipids extracted by different methods

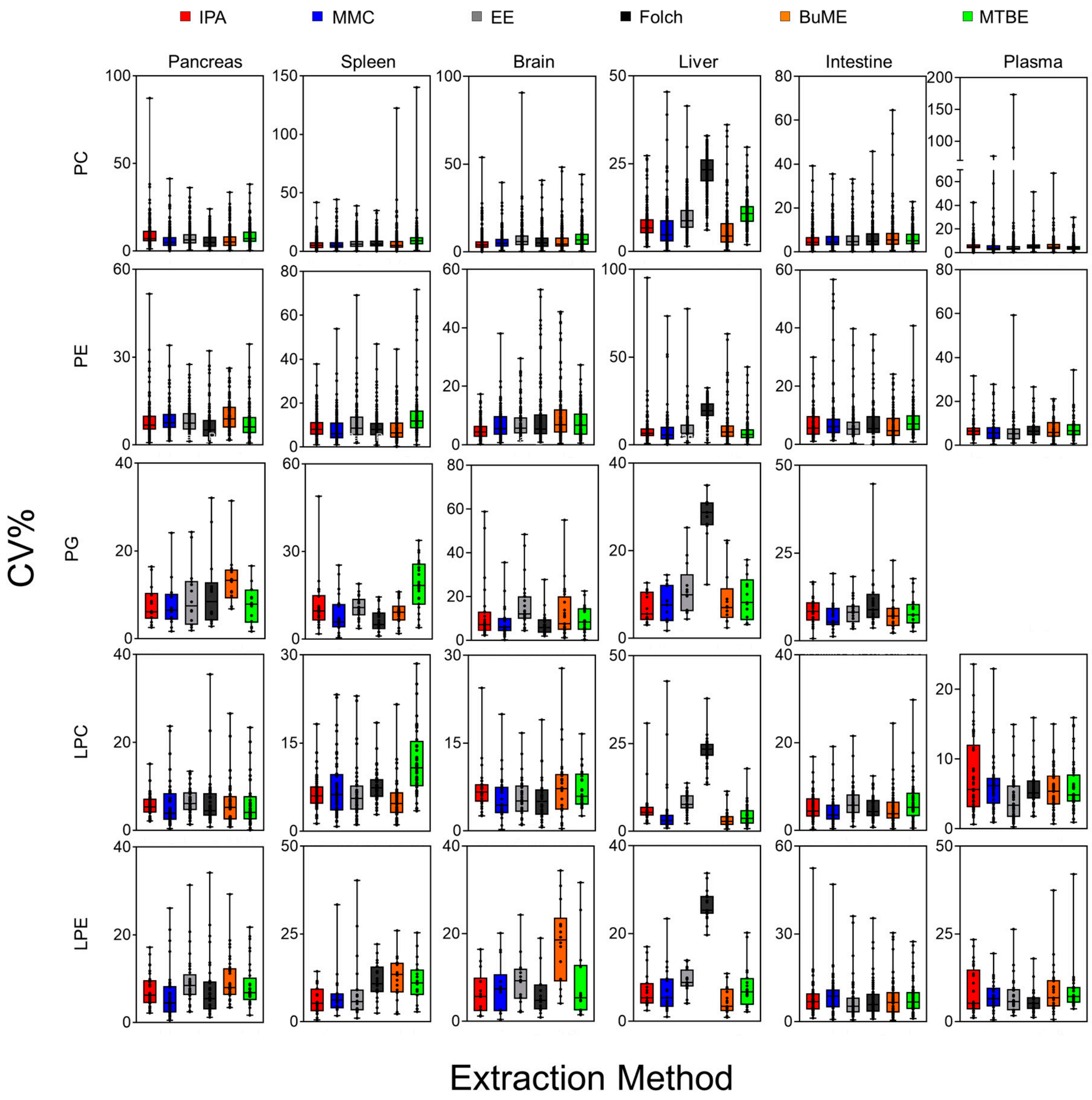

Figure S2. The inter-assay coefficient of variation (CV%) of absolute concentrations of endogenous glycerophospholipids extracted by different methods

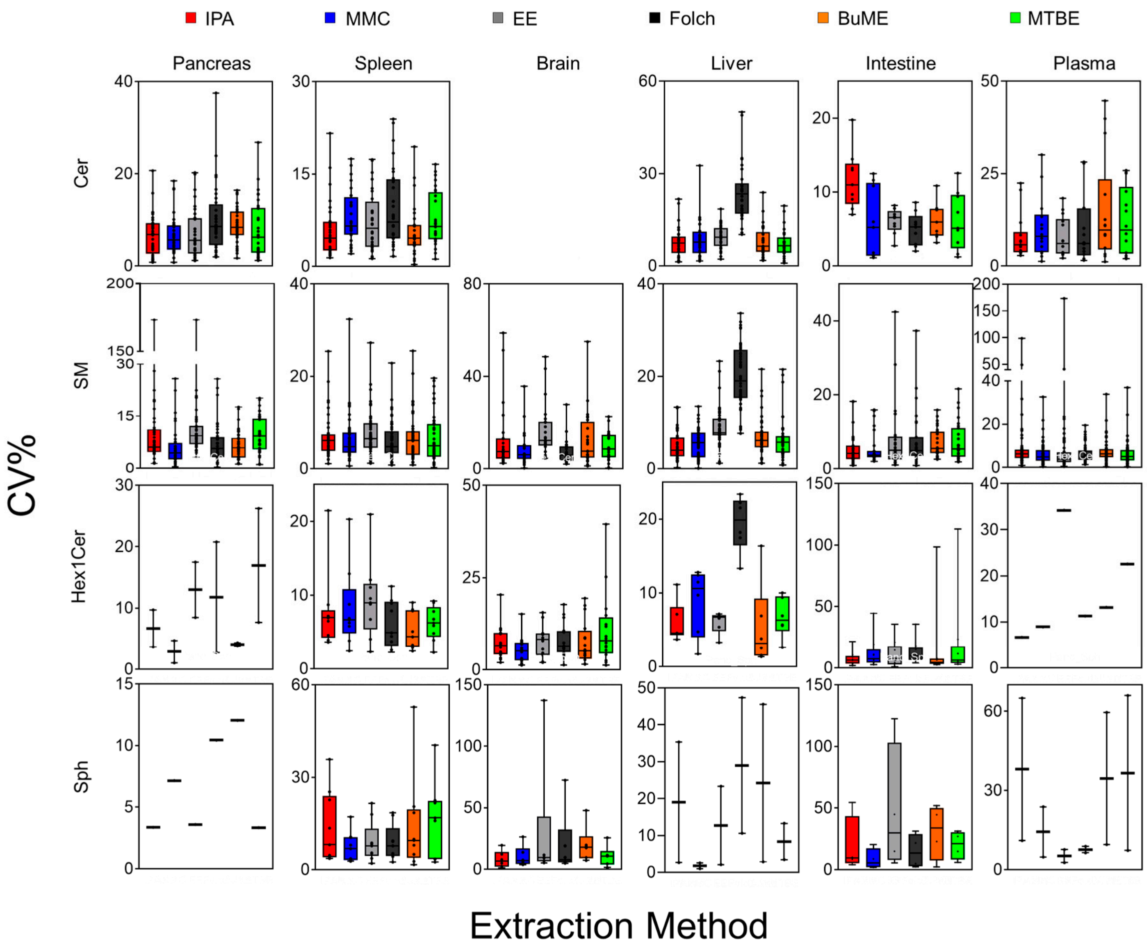

Figure S3. The inter-assay coefficient of variation (CV%) of absolute concentrations of endogenous sphingolipids extracted by different methods

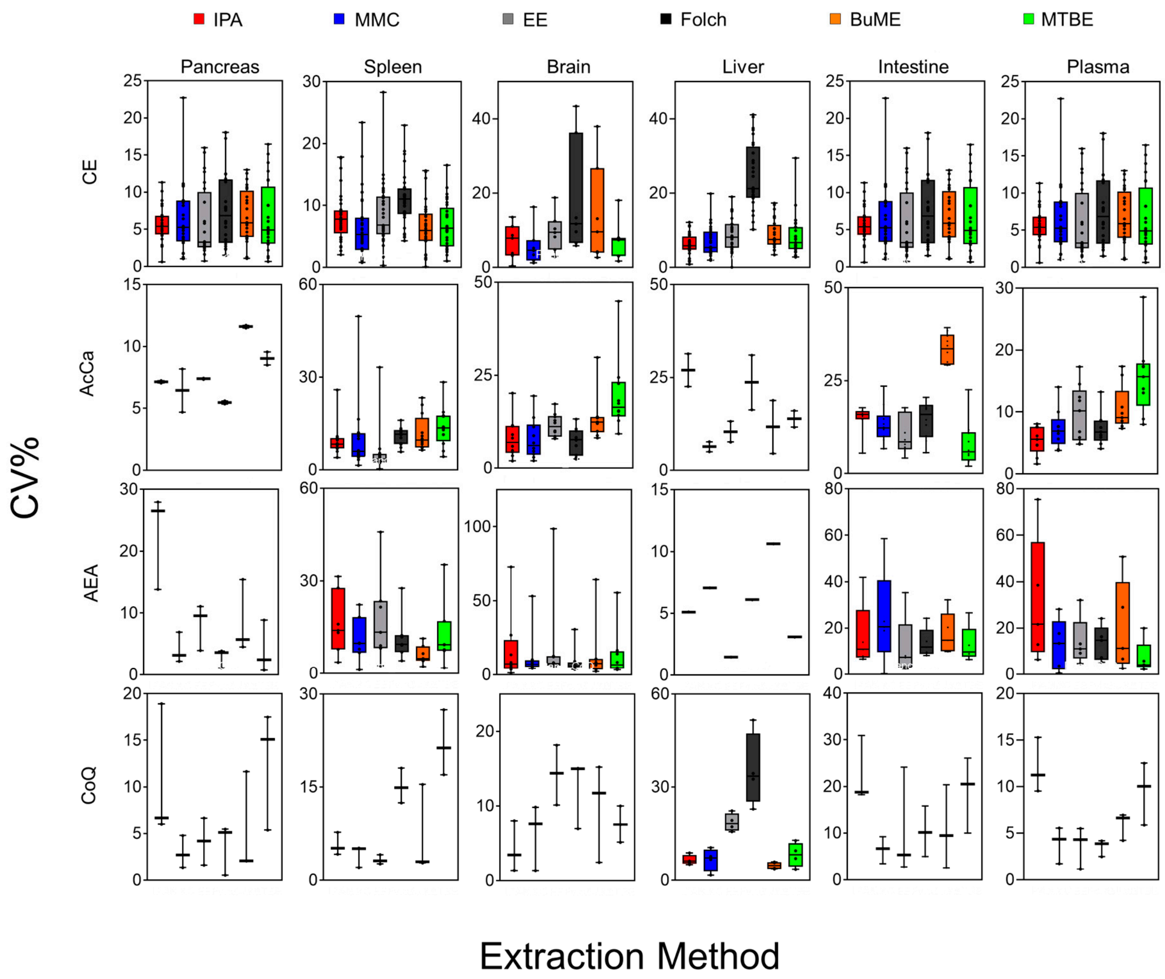

Figure S4. The inter-assay coefficient of variation (CV%) of absolute concentrations of endogenous CE, AcCa, AEA, and CoQ extracted by different methods
